
JOURNAL INFORMATION
==============================
NLM Title Abbreviation: Tex Med J (Austin)
NLM Journal ID: 21130590R

Texas Medical Journal

ISSN: 0892-8495
Publisher: Biomedical Journal Digitization Project

ARTICLE INFORMATION
==============================
PMCID: PMC9591689
Article version: 1
Subjects: Books and Magazines

In the Wake of the War, a Novel

Print publication date: 1900 Nov
Volume: 16
Issue: 5
First page: 223
Last page: 223
License: This work is free of known copyright restrictions. For more information, please see PMC Back Issue Digitization.
License URL: https://creativecommons.org/publicdomain/mark/1.0/

==============================

D.

---

IN THE WAKE OF THE WAR, a novel. By Verne S. Pease, Chicago. Geo. M. Hill Co., Publishers. Cloth, 12mo, 440 pages, tastefully printed and attractively bound. Price, $1.25 postpaid.

This is a stirring story of the days of "reconstruction," under carpet-bag rule in the South, when the "Freedmans' Bureau" was in bloom, and the Kuklux Klan was its fruitage. It is told in a pleasing manner, and has a peculiar interest to those who like the writer of this were witnesses to many such occurrences as those described by Mr. Pease. It will be of interest also to the younger generations, as throwing a strong side light on the history of that turbulent and trying period just after the real fighters had laid down their arms and shaken hands.

---

### Publisher's Department.

"I PRESCRIBE Hagee's Cordial of Cod Liver Oil Compound as a palatable up-builder."

A. M. CARPENTER, M. D.,
Professor of the Principles and Practice of Medicine and Clinical Medicine, Barnes' Medical College, 3537 Lindell Ave., St. Louis.

---

"I HAVE been prescribing Hagee's Cordial Cod Liver Oil Compound for years with very satisfactory results in many diseases where reconstructives and nutritives are indicated, as well as insipient phthisis and obscure diseases. On account of its palatability patients will take it in quantities and long enough to secure results."

G. W. BUCHANAN, M. D., Richmond, Mo.
